# Omission of axillary surgery for ipsilateral breast tumor recurrence with negative nodes after previous breast-conserving surgery: is it oncologically safe?

**DOI:** 10.1007/s10549-022-06708-y

**Published:** 2022-08-30

**Authors:** Fei-Lin Qu, Cai-Jin Lin, Zhe-Bin Liu, A.-Yong Cao, Jiong Wu, Guang-Yu Liu, Ke-Da Yu, Gen-Hong Di, Jun-Jie Li, Zhi-Ming Shao

**Affiliations:** 1 Department of Breast Surgery, Fudan University Shanghai Cancer Center, Shanghai, 200032 China; 2 Department of Oncology, Shanghai Medical College, Fudan University, Shanghai, 200032 China; 3 Key Laboratory of Breast Cancer in Shanghai, Fudan University Shanghai Cancer Center, Shanghai, 200032 China

**Keywords:** Breast-conserving surgery, Ipsilateral breast tumor recurrence, Surgical axillary staging, Breast cancer

## Abstract

**Purpose:**

Salvage mastectomy is traditionally recommended for patients who developed ipsilateral breast tumor recurrence (IBTR) in light of previous breast irradiation. However, it remains controversial whether surgical axillary staging (SAS) is necessary for IBTR patients with negative nodes. This study aimed to evaluate the oncologic safety of omitting SAS for IBTR.

**Methods:**

We retrospectively identified patients who developed invasive IBTR with negative nodes after undergoing breast-conserving surgery (BCS) from 2010 to 2018. Patterns of care in nodal staging were analyzed based on prior axillary staging status. Clinicopathologic characteristics and adjuvant treatment of the initial tumor, as well as the IBTR, were compared between the SAS and no SAS groups. Kaplan–Meier method and Cox regression model were utilized to compare the locoregional recurrence-free survival (LRRFS), distant metastasis-free survival (DMFS), and overall survival (OS) rates after IBTR removal between the two groups.

**Results:**

A total of 154 IBTR patients were eligible for final analysis. Compared to the no SAS group, SAS group was less likely to undergo ALND (15.1 vs 73.3%, *p* < 0.001) at initial BCS, had a longer recurrence interval (2.8 vs 2.1 years, *p* = 0.03), and were more likely to have discordant molecular subtype (35.8 vs 12.9%, *p* = 0.001) and different quadrant location (37.7 vs 19.8%, *p* = 0.02) with primary tumor. However, the extent of axillary staging did not affect systemic or radiation recommendations. In the subgroup of patients without previous ALND, the clinicopathologic characteristics were roughly comparable. No significant differences were observed in LRRFS, DMFS or OS between the two groups.

**Conclusion:**

For node-negative IBTR patients, we observed selection bias on the basis of prior ALND, shorter recurrence interval, and concordant molecular subtype favoring no SAS but comparable LRRFS, DMFS, and OS. These results support a wider consideration of sparing SAS in the management of IBTR, especially in patients without previous ALND.

**Supplementary Information:**

The online version contains supplementary material available at 10.1007/s10549-022-06708-y.

## Introduction

Even with routine practice of breast-conserving surgery (BCS) and adjuvant radiotherapy, ipsilateral breast tumor recurrence (IBTR) accounts for 5–15% of all cancer recurrence in patients with early-stage breast cancer (EBC) [1, 2]. The standard of care for IBTR is salvage mastectomy in light of previous radiation treatment [3]. Repeat BCS with reirradiation could be an alternative to mastectomy in some highly selected patients [4]

However, the optimal axillary management in patients with IBTR is still under debate, particularly in those who have been previously treated with sentinel lymph node biopsy (SLNB). In the 2021 St. Gallen consensus voting, repeat attempts at SLNB were particularly favored by the Panel in the setting of IBTR patients with negative nodes on imaging after previous treatment with negative sentinel node mapping [5]. In contrast, the voting results of the Chinese Anti-Cancer Association Committee of Breast Cancer Society guideline (CBCS guideline) panelists were split 50/50 on offering completion axillary lymph node dissection (ALND) instead of repeat SLNB.

In primary breast cancer, the value of nodal staging and regional disease control after SLNB without ALND has been evaluated in several randomized controlled trials [6, 7]. These results have demonstrated that many patients with EBC can be spared the morbidity related to axillary clearance without an increased risk of regional recurrence or impacting breast cancer-specific survival. While nodal status in primary breast cancer is associated with prognosis and predictive information, the role of axillary staging in recurrence is still unclear. Some studies have reported the feasibility of repeat SLNB in patients with IBTR [8, 9], while others have more recently questioned its value for patients who are at sufficient risk of developing relapse that will require systemic therapies regardless of nodal status [10]. Nonetheless, long-term follow-up data on regional recurrence after less extensive axillary treatment have not yet been available. For the omission of SAS to become an alternative in IBTR scenario, it is imperative to ensure high regional disease control.

Therefore, by retrospectively reviewing our institution-based database, we were able to describe patterns of care in axillary staging management for IBTR. The first aim of this study was to assess the safety profile of omitting SAS for IBTR in terms of locoregional recurrence-free survival (LRRFS). The second aim was to evaluate whether axillary surgery would impact adjuvant treatment recommendations. Furthermore, this study identified tumor-related factors associated with axillary surgery in IBTR patients.

## Materials and methods

### Patients

**Fig. 1:**
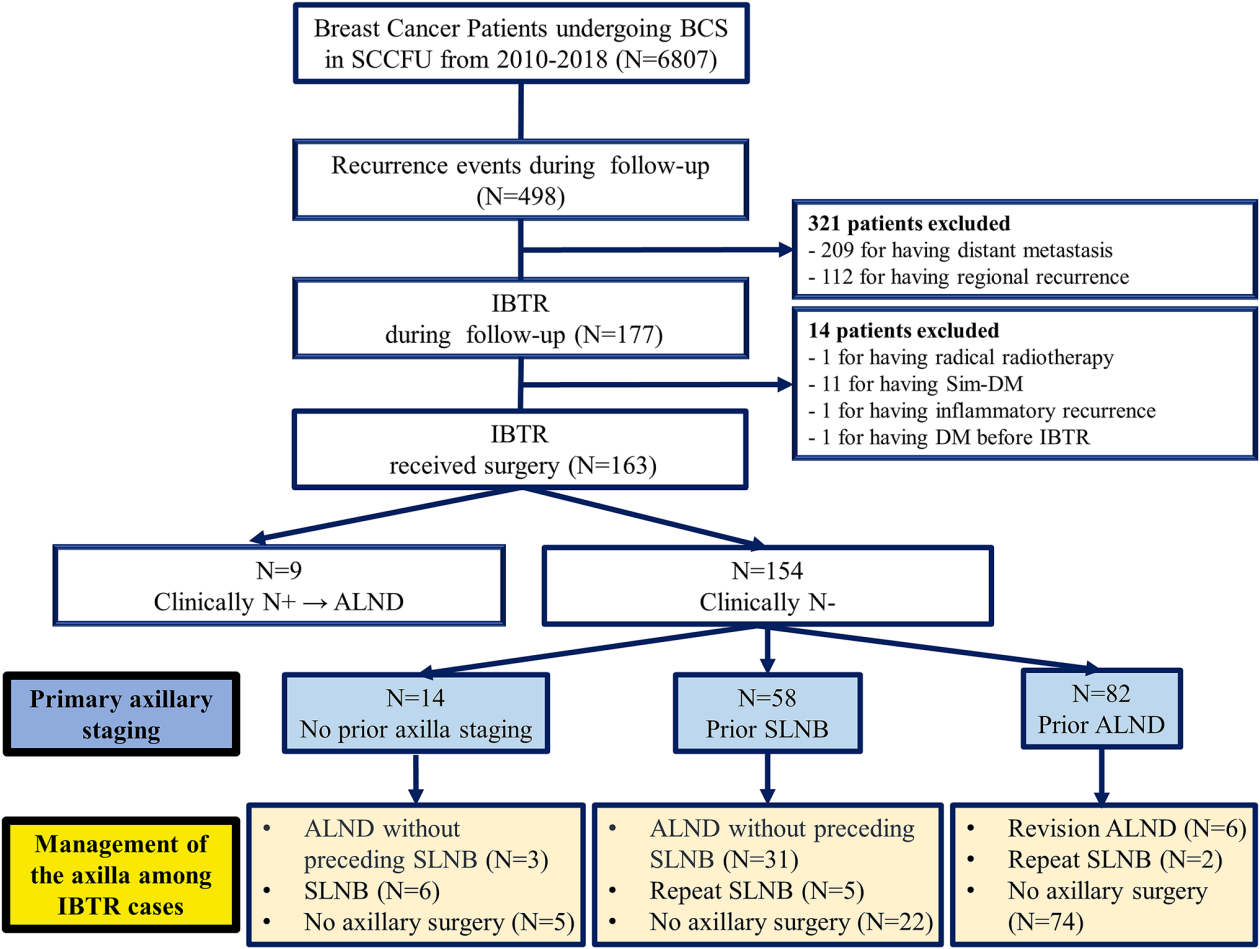
Flow diagram of axillary staging management for IBTR patients with negative nodes. *BCS* breast-conserving surgery, *SCCFU* Shanghai Cancer Center, Fudan University, *IBTR* ipsilateral breast tumor recurrence, *Sim-DM* simultaneous distant metastasis, *DM* distant metastasis, *N* + positive lymph nodes, *ALND* axillary lymph node dissection, *N-* negative lymph node, *SLNB* sentinel lymph node biopsy

All consecutive patients with breast cancer undergoing surgery from Jan 2010 to Dec 2018 were retrospectively retrieved. All clinical data were sourced from a hospital-based cancer registry database as previously described [11]. The inclusion criteria were as follows: (1) histologically diagnosed invasive breast cancer patients; (2) initially treated with breast-conserving surgery; (3) occurrence of isolated invasive in-breast recurrence with ipsilateral clinically negative axillary lymph node (cN0, including negative clinical examination and no suspicious lymph nodes detected by ultrasound) as the first event during follow-up; (4) histopathological analysis of recurrent/metastatic lesions by resection. We excluded patients with in situ IBTR or non-resected IBTR, with prior neoadjuvant chemotherapy, with a diagnosis of bilateral breast cancer, with ipsilateral clinically positive axillary lymph node (cN + , including positive clinical examination or suspicious/metastatic lymph nodes detected by ultrasound) or with concurrent distant metastasis at time of IBTR detection (Fig. 1). Data regarding the prior cancer diagnosis were recorded, including age at diagnosis, nodal and size staging, grade, molecular subtype, details regarding primary axillary surgery and adjuvant therapies. With respect to patient and treatment data at the time of IBTR, details regarding recurrence interval were collected along with surgical treatment of breast and axilla, as were the age at diagnosis, nodal and size staging, grade, molecular subtype concordance, focality of IBTR, location of IBTR, and subsequent treatment. This study was approved by the independent Ethical Committees of Shanghai Cancer Center, Fudan University and was in accordance with the Helsinki Declaration. Patient consent to review their medical records was waived due to the retrospective nature of this study. Meanwhile, patients included were anonymous, and all medical data of the patients were kept confidential.

### Diagnosis and treatment

In this study, IBTR was defined as an invasive carcinoma that occurred after BCS in the ipsilateral breast without clinical-radiologic evidence of regional disease, and no distinction was further analyzed between the two types of IBTR—true recurrence and new primary tumor [12, 13]. The procedure of the BCS technique in our center was described previously [11]. After initial BCS, each patient was recommended to undergo standard treatments, including chemotherapy, irradiation, anti-HER2 therapy, and endocrine therapy, alone or in combination. Local treatment of IBTR consisted of repeat breast-conserving treatment, including reirradiation (typically partial breast) or salvage mastectomy with or without irradiation of the chest wall and regional nodal areas (supra/infraclavicular + internal mammary). No intentional axillary irradiation was prescribed according to the clinical target volume in our center protocols [14]. Adjuvant systemic treatments following IBTR resection were determined by a multidisciplinary team of breast cancer experts informed by the tumor biology of the initial/recurrent tumors, and previous treatments.

### Definition of study endpoints

The primary endpoint of this study was locoregional recurrence (LRR) as the first event even after curative treatment of IBTR, including any evidence of disease found in the ipsilateral chest wall, supra/infraclavicular nodes, internal mammary nodes, and axillary nodes. The secondary endpoints of this study were distant metastasis-free survival (DMFS) and overall survival (OS). LRRFS was defined as the interval between the date of the diagnosis of IBTR and the date of any evidence found in LRR. DMFS was defined as the interval between the date of IBTR and the date of distant metastasis. OS was defined as the interval between the date of IBTR and the date of death from any cause. In cases of synchronous LRR and distant metastasis, recurrence was registered as both events.

### Statistical analysis

Patient and tumor characteristics were summarized using descriptive statistics. Statistical significance was calculated using the Pearson chi square test and Fisher’s exact test for categorical variables (excluding unknown values). Mann–Whitney *U* test or independent samples T test was utilized for continuous variables. Kaplan–Meier method was used to determine the LRRFS, DMFS, and OS. The LRRFS, DMFS, and OS data were presented using the Cox regression model. The significance of the survival differences was calculated using the log-rank test. All *p* values were two sided, and a *p* value of < 0.05 was considered statistically significant. Statistical analyses were carried out using R version 3.4.1 (http://www.R-project.org) with its appropriate packages and Statistical Package for Social Sciences (version 26.0) software (SPSS Inc., Chicago, IL, USA).

## Results

A total of 177 (2.6%) of 6807 patients undergoing BCS from 2010 to 2018 were diagnosed with IBTR. Clinical data from 163 patients were available for review. Nine patients with cN + underwent ALND at time of IBTR; hence, the remaining 154 patients were available for final analysis. Of the 154 included patients, 82 (53.2%) underwent ALND without preceding SLNB, 58 (37.7%) were offered SLNB with all negative nodes, and 14 (9.1%) did not receive any axillary surgery related to the primary tumor. The distribution of axillary staging status for IBTR is detailed in Fig. 1.

### Patterns of care in axilla nodal staging in IBTR patients with negative nodes

**Fig. 2:**
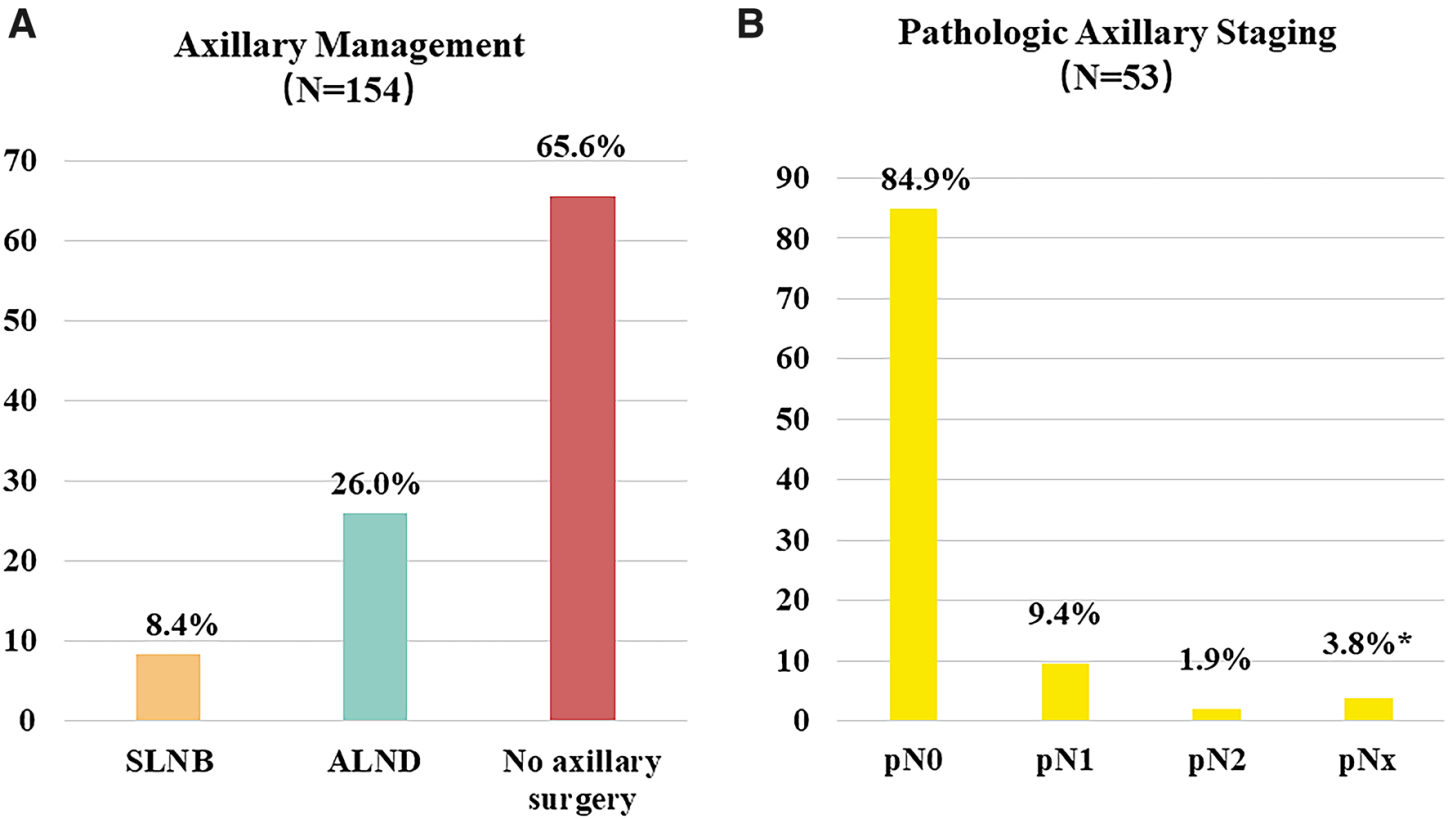
Axillary management in all patients with negative-node IBTR (N = 154). **A** Distribution of axillary management. **B** Pathologic axillary staging in patients receiving axillary surgery at time of IBTR. *SLNB* sentinel lymph node biopsy, *ALND* axillary lymph node dissection *pNx, two patients received repeat SLNB after prior SLNB or ALND, but with unsuccessful sentinel node identification

Among all 154 IBTR patients with cN0 at time of IBTR, axillary surgery was not performed in 101 (65.6%) patients. The remaining 53 (34.4%) patients underwent either repeat SLNB or ALND without preceding SLNB, accounting for 13 (8.4%) and 40 (26.0%) cases, respectively (Fig. 2A). Of the 53 patients who underwent SAS, 45 (84.9%) were pathologically node-negative (Fig. 2B), and 2 (3.8%) had unknown node status for unsuccessful repeat sentinel node identification (Supplementary Table 1).

**Fig. 3:**
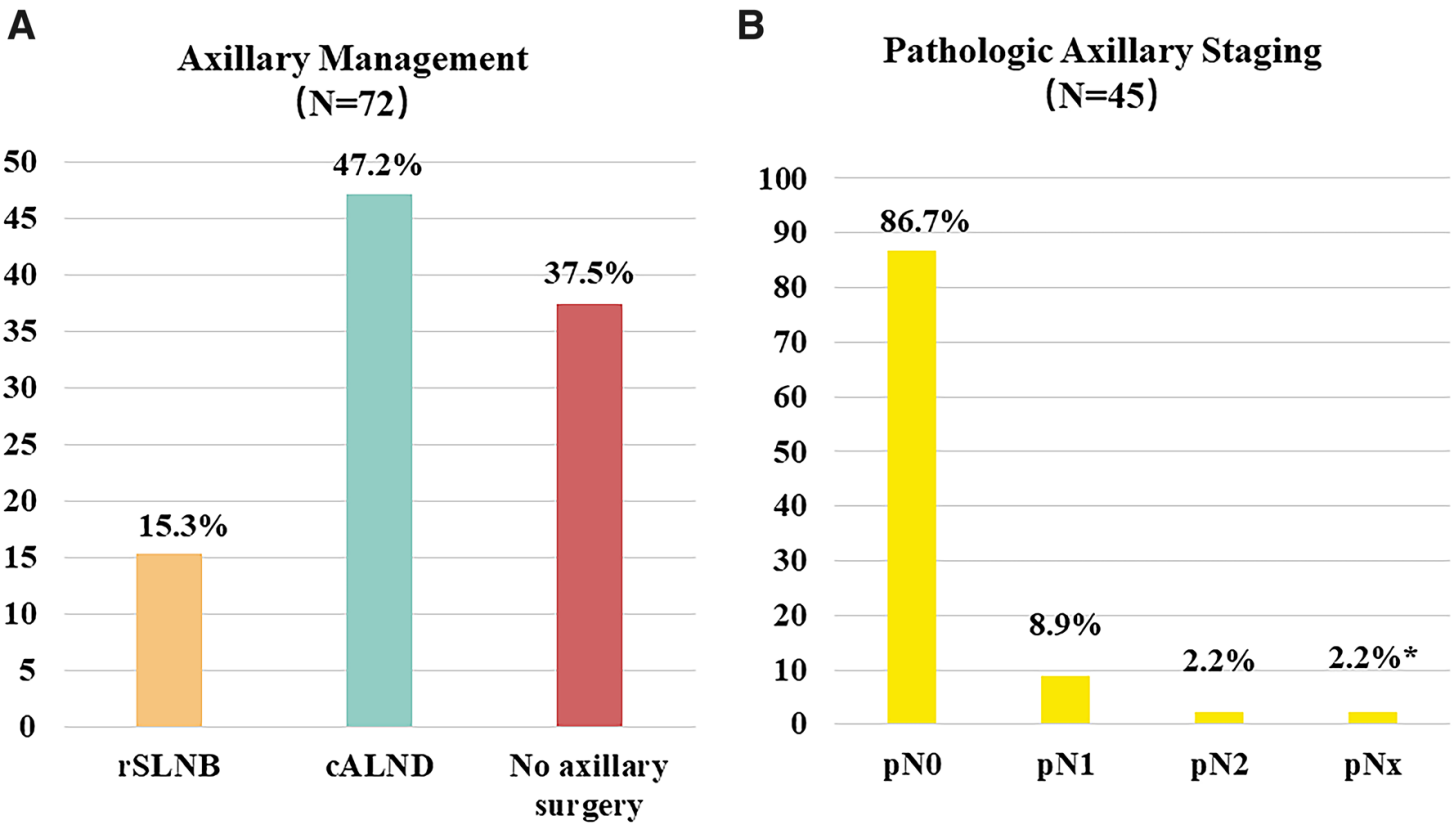
Axillary management in patients without prior ALND and negative-node IBTR (*N* = 72). **A** Distribution of axillary management. **B** Pathologic axillary staging in patients receiving axillary surgery at time of IBTR. *rSLNB* repeat sentinel lymph node biopsy, *cALND* completion axillary lymph node dissection. *pNx, one patient received repeat SLNB after prior SLNB, but with unsuccessful sentinel node identification

Moreover, analyses stratified by prior axillary staging status showed that almost half (47.2%) of patients with no previous ALND were offered ALND without preceding SLNB as SAS at time of IBTR (Fig. 3A). Of the 45 patients who underwent SAS, 39 (86.7%) had pathologically negative nodal status, concordant with that of the whole cohort (Fig. 3B).

### Factors related to the performance of surgical axillary staging in IBTR patients with negative nodes

Although the initial tumor characteristics for axilla surgery vs no axilla surgery at time of IBTR were roughly comparable in the overall population (Table 1), patients treated by axilla surgery were less likely to have undergone previous ALND (15.1% vs 73.3%, *p* < 0.001), with a lower percentage of nodal involvement related to the primary tumor (11.3% vs 44.6%, *p* < 0.001), and were less likely to receive chemotherapy for the primary tumor (60.4% vs 80.2%, *p* = 0.01). We did not find other significant differences between the two groups regarding age at initial diagnosis, tumor size, tumor grade, or molecular subtype of primary breast cancer. Additionally, there were no significant differences in the administration of radiotherapy, anti-HER2 therapy, and endocrine therapy for primary breast cancer between the two groups (60.4% vs 64.4%, 18.9% vs 11.9%, and 34.0% vs 34.7%, *p* = 0.63, 0.24, and 0.93, respectively).

**Table 1:** Clinicopathological characteristics of all patients at primary tumor and IBTR diagnosis (*N* = 154)

	Total patients with IBTR (*N* = 154, %)	Axilla surgery for IBTR (*N* = 53, %)	No axilla surgery for IBTR (*N* = 101, %)	*p* value
Primary tumor				
Age primary tumor, median years(range)	43 (21–84)	42 (27–82)	43 (21–84)	0.84
Primary axilla surgery				< 0.001
No axilla staging	14 (9.1)	9 (17.0)	5 (5.0)	
SLNB	58 (37.7)	36 (67.9)	22 (21.7)	
ALND	82 (53.2)	8 (15.1)	74 (73.3)	
Pathologic nodal status (primary tumor)				< 0.001
Negative	89 (57.8)	38 (71.7)	51 (50.5)	
Positive	51 (33.1)	6 (11.3)	45 (44.6)	
Unknown	14 (9.1)	9 (17.0)	5 (4.9)	
Pathologic tumor size (primary tumor, mm)				0.40
≤ 20	91 (59.1)	33 (62.3)	58 (57.4)	
21–50	38 (24.7)	12 (22.6)	26 (25.7)	
> 50	3 (1.9)	0 (0)	3 (3.0)	
Unknown	22 (14.3)	8 (15.1)	14 (13.9)	
Primary tumor grade				0.73
I	1 (0.6)	0 (0)	1 (1.0)	
II	56 (36.4)	15 (28.3)	41 (40.6)	
III	55 (35.7)	17 (32.1)	38 (37.6)	
Unknown	42 (27.3)	21 (39.6)	21 (20.8)	
Molecular subtype of primary tumor				0.56
HR + /HER2-	57 (37.0)	21 (39.6)	36 (35.6)	
HR + /HER2 +	19 (12.3)	4 (7.4)	15 (14.9)	
HR-/HER2 +	33 (21.5)	13 (24.5)	20 (19.8)	
HR-/HER2-	43 (27.9)	14 (26.6)	29 (28.7)	
Unknown	2 (1.3)	1 (1.9)	1 (1.0)	
Radiotherapy for primary tumor				0.63
Yes	97 (63.0)	32 (60.4)	65 (64.4)	
No	57 (37.0)	21 (39.6)	36 (35.6)	
Chemotherapy for primary tumor				0.01
Yes	113 (73.4)	32 (60.4)	81 (80.2)	
No	41 (26.6)	21 (39.6)	20 (19.8)	
Anti-HER2 therapy for primary tumor				0.24
Yes	22 (14.3)	10 (18.9)	12 (11.9)	
No	132 (85.7)	43 (81.1)	89 (88.1)	
Endocrine therapy for primary tumor				0.93
Yes	53 (34.4)	18 (34.0)	35 (34.7)	
No	101 (65.6)	35 (66.0)	66 (65.3)	
IBTR tumor				
Age IBTR, median years(range)	46 (21–84)	46 (28–83)	48 (26–88)	0.53
Time from primary tumor to IBTR diagnosis				
Median, years (IQR)	2.6 (1.3–4.2)	2.8 (1.3–5.0)	2.1 (1.3–3.5)	0.03
IBTR surgery				0.40
Mastectomy	138 (89.6)	49 (92.5)	89 (88.1)	
RCT	16 (10.4)	4 (7.5)	12 (11.9)	
Pathologic nodal status (IBTR)				N/A
Negative	45 (29.2)	45 (84.9)	N/A	
Positive	6 (3.9)	6 (11.3)	N/A	
Unknown	103 (66.9)	2^#^ (3.8)	101 (100)	
Pathologic tumor size (IBTR, mm)				0.55
≤ 20	90 (58.4)	29 (54.7)	61 (60.4)	
21–50	38 (24.7)	16 (30.2)	22 (21.8)	
> 50	5 (3.2)	2 (3.8)	3 (3.0)	
Unknown	21 (13.7)	6 (11.3)	15 (14.8)	
IBTR tumor grade				0.56
I	4 (2.6)	2 (3.8)	2 (2.0)	
II	48 (31.1)	18 (34.0)	30 (29.7)	
III	60 (39.0)	18 (34.0)	42 (41.6)	
Unknown	42 (27.3)	15 (28.2)	27 (26.7)	
Molecular subtype of IBTR				0.001
Concordant (with primary tumor)	118 (76.6)	32 (60.4)	86 (85.1)	
Discordant	32 (20.8)	19 (35.8)	13 (12.9)	
Unknown	4 (2.6)	2 (3.8)	2 (2.0)	
Location of IBTR (quadrant)				0.02
Within same quadrant	114 (74.0)	33 (62.3)	81 (80.2)	
Outside treatment field	40 (26.0)	20 (37.7)	20 (19.8)	
Chemotherapy for IBTR (adjuvant)				0.16
Yes	102 (66.2)	39 (73.6)	63 (62.4)	
No	52 (33.8)	14 (26.4)	38 (37.6)	
Radiotherapy for IBTR				0.32
Yes	30* (19.5)	8 (15.1)	22 (21.8)	
No	124 (80.5)	45 (84.9)	79 (78.2)	
Anti-HER2 therapy for IBTR (adjuvant)				0.85
Yes	45 (29.2)	16 (30.2)	29 (28.7)	
No	109 (70.8)	37 (69.8)	72 (71.3)	
Endocrine therapy for IBTR (adjuvant)				0.50
Yes	55 (35.7)	17 (32.1)	38 (37.6)	
No	99 (64.3)	36 (67.9)	63 (62.4)	

*IBTR* ipsilateral breast tumor recurrence, *IQR* interquartile range, *SLNB* sentinel lymph node biopsy, *ALND* axilla lymph node dissection, *RCT* repeat conservation treatment, *HR* hormone receptor

^#^Two patients received repeat SLNB after prior SLNB or ALND, but with unsuccessful sentinel node identification

*Of all patients recommended with irradiation following IBTR surgery, twenty (66.7%) of them did not receive radiation therapy after previous breast-conserving surgery. The remaining 10 patients with previous irradiation were treated with curative operation of IBTR followed by second irradiation

As shown in Supplementary Fig. 1, transitions between clinicopathologic features from primary tumor to IBTR were common. Compared to those undergoing no axilla surgery, patients undergoing axilla surgery had a significantly longer time interval from primary cancer to IBTR (median: 2.8 years, IQR (1.3–5.0) vs 2.1 years, IQR (1.3–3.5), *p* = 0.03), and were more likely to have discordant molecular subtypes (35.8% vs 12.9%, *p* = 0.001) and different quadrant location (37.7% vs 19.8%, *p* = 0.02) with the primary tumor. No significant differences were found between the two groups in the age at IBTR diagnosis, tumor size, tumor grade, regional nodal status, IBTR surgical treatment, or molecular subtype for IBTR. Concordantly, no significant difference was observed in the administration of adjuvant therapies for IBTR regardless of axillary staging status. Of note, radiotherapy after IBTR resection was performed in 30 (19.5%) patients, of whom twenty (66.7%) did not receive adjuvant radiation therapy after previous BCS. Chemotherapy, anti-HER2 therapy, and endocrine therapy for IBTR were prescribed in 73.6% vs 62.4% (*p* = 0.16), 30.2% vs 28.7% (*p* = 0.85), and 32.1% vs 37.6 (*p* = 0.50) of the two groups, respectively.

Furthermore, analysis stratified by axillary staging status of primary breast cancer demonstrated certain inconsistencies with the results of the whole cohort (Table 2). No significant differences were observed in the prior axillary staging status (*p* = 0.88), IBTR interval (2.1 years vs 1.8 years, *p* = 0.68), IBTR molecular subtype (discordant with primary tumor: 26.7% vs 11.1%, *p* = 0.11), or IBTR location (different quadrant location: 31.1% vs 22.2%, *p* = 0.42) between the axilla surgery and no axilla surgery groups in patients with no previous ALND.

**Table 2:** Clinicopathological characteristics of IBTR patients with no previous ALND (*N* = 72)

	Patients without prior ALND (*N* = 72%)	Axilla surgery for IBTR (*N* = 45%)	No axilla surgery for IBTR (*N* = 27%)	*p* value
Primary tumor				
Age primary tumor, median years(range)	42.5 (21–82)	43 (27–82)	41 (21–78)	0.64
Primary axilla surgery				0.88
No axilla staging	14 (19.4)	9 (20.0)	5 (18.5)	
SLNB	58 (80.6)	36 (80.0)	22 (81.5)	
Pathologic nodal status (primary tumor)				0.88
Negative	58 (80.6)	36 (80.0)	22 (81.5)	
Unknown	14 (19.4)	9 (20.0)	5 (18.5)	
Pathologic tumor size (primary tumor, mm)				0.65
≤ 20	48 (66.6)	30 (66.7)	18 (66.7)	
> 20	13 (18.1)	9 (20.0)	4 (14.8)	
Unknown	11 (15.3)	6 (13.3)	5 (18.5)	
Primary tumor grade				0.44
I	1 (1.4)	0 (0)	1 (3.7)	
II	19 (26.4)	12 (26.7)	7 (25.9)	
III	25 (34.7)	14 (31.1)	11 (40.8)	
Unknown	27 (37.5)	19 (42.2)	8 (29.6)	
Molecular subtype of primary tumor				0.74
HR + /HER2-	29 (40.3)	19 (42.2)	10 (37.0)	
HR + /HER2 +	7 (9.7)	4 (8.9)	3 (11.1)	
HR-/HER2 +	16 (22.2)	12 (26.7)	4 (14.9)	
HR-/HER2-	19 (26.4)	10 (22.2)	9 (33.3)	
Unknown	1 (1.4)	0 (0)	1 (3.7)	
Radiotherapy for primary tumor				0.27
Yes	38 (52.8)	26 (57.8)	12 (44.4)	
No	34 (47.2)	19 (42.2)	15 (55.6)	
Chemotherapy for primary tumor				0.67
Yes	37 (51.4)	24 (53.3)	13 (48.1)	
No	35 (48.6)	21 (46.7)	14 (51.9)	
Anti-HER2 therapy for primary tumor				0.86
Yes	10 (13.9)	6 (13.3)	4 (14.8)	
No	62 (86.1)	39 (86.7)	23 (85.2)	
Endocrine therapy for primary tumor				0.61
Yes	24 (33.3)	16 (35.6)	8 (29.6)	
No	48 (66.7)	29 (64.4)	19 (70.4)	
IBTR tumor				
Age IBTR, median years(range)	45 (23–82)	46 (28–82)	42 (23–81)	0.70
Time from primary tumor to IBTR diagnosis				0.68
Median, years (IQR)	2.0 (1.0–3.5)	2.1 (1.3–3.5)	1.8 (0.75–3.6)	
IBTR breast surgery				0.12
Mastectomy	64 (88.9)	42 (93.3)	22 (81.5)	
RCT	8 (11.1)	3 (6.7)	5 (18.5)	
Pathologic nodal status (IBTR)				N/A
Negative	39 (54.2)	39 (86.7)	N/A	
Positive	5 (6.9)	5 (11.1)	N/A	
Unknown	28 (38.9)	1^#^ (2.2)	27 (100)	
Pathologic tumor size (IBTR, mm)				0.59
≤ 20	50 (69.4)	32 (71.1)	18 (66.7)	
> 20	21 (29.2)	12 (26.7)	9 (33.3)	
Unknown	1 (1.4)	1 (2.2)	0 (0)	
IBTR tumor grade				0.46
I	2 (2.8)	2 (4.4)	0 (0)	
II	24 (33.3)	15 (33.4)	9 (33.3)	
III	25 (34.7)	14 (31.1)	11 (40.8)	
Unknown	21 (29.2)	14 (31.1)	7 (25.9)	
Molecular subtype of IBTR				0.11
Concordant (with primary tumor)	56 (77.8)	32 (71.1)	24 (88.9)	
Discordant	15 (20.8)	12 (26.7)	3 (11.1)	
Unknown	1 (1.4)	1 (2.2)	0 (0)	
Location of IBTR (quadrant)				0.42
Within same quadrant	52 (72.2)	31 (68.9)	21 (77.8)	
Outside treatment field	20 (27.8)	14 (31.1)	6 (22.2)	
Chemotherapy for IBTR (adjuvant)				0.74
Yes	46 (63.9)	30 (66.7)	16 (59.3)	
No	20 (27.8)	12 (26.7)	8 (29.6)	
Unknown	6 (8.3)	3 (6.6)	3 (11.1)	
Radiotherapy for IBTR				0.24
Yes	15* (20.8)	7 (15.6)	8 (29.6)	
No	51 (70.8)	35 (77.8)	16 (59.3)	
Unknown	6 (8.4)	3 (6.6)	3 (11.1)	
Anti-HER2 therapy for IBTR (adjuvant)				0.37
Yes	20 (27.8)	15 (33.3)	5 (18.5)	
No	46 (63.9)	27 (60.0)	19 (70.4)	
Unknown	6 (8.3)	3 (6.7)	3 (11.1)	
Endocrine therapy for IBTR (adjuvant)				0.72
Yes	25 (34.7)	15 (33.3)	10 (37.0)	
No	41 (57.0)	27 (60.0)	14 (51.9)	
Unknown	6 (8.3)	3 (6.7)	3 (11.1)	

*IBTR* ipsilateral breast tumor recurrence, *IQR* interquartile range, *SLNB* sentinel lymph node biopsy, *ALND* axilla lymph node dissection, *RCT* repeat conservation treatment, *HR* hormone receptor

^#^One patient received repeat SLNB after prior SLNB, but with unsuccessful sentinel node identification

*Of all patients recommended with irradiation following IBTR surgery, thirteen (86.7%) of them did not receive radiation therapy after previous breast-conserving surgery. The remaining 2 patients with previous irradiation were treated with curative operation of IBTR followed by second irradiation

### Regional and distant recurrence after curative treatment of IBTR

**Fig. 4:**
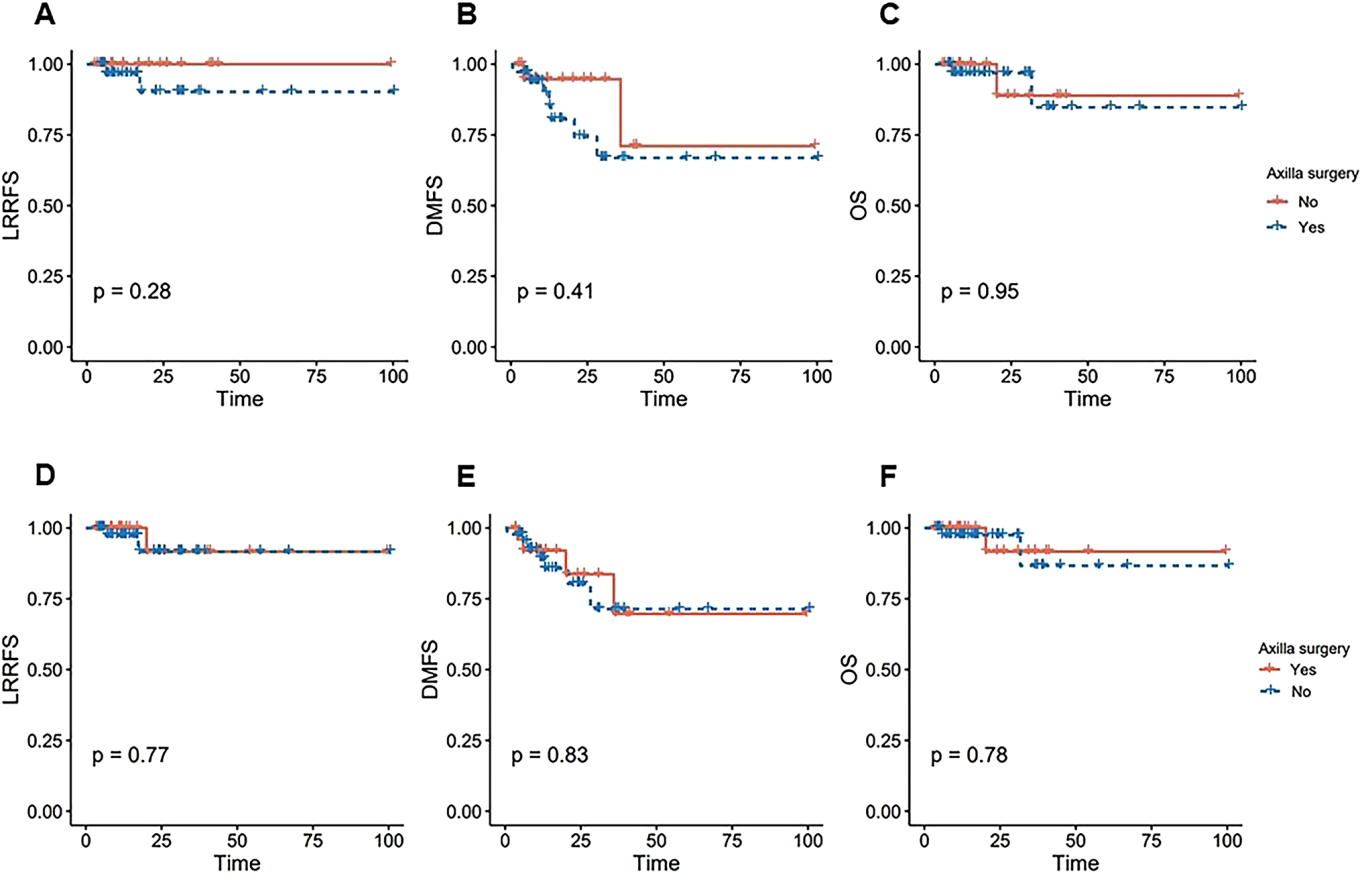
(**A**–**C**) LRRFS, DMFS, and OS between axilla surgery and no axilla surgery groups in IBTR patients with negative nodes (*N* = 154); (**D**–**F**) LRRFS, DMFS, and OS between axilla surgery and no axilla surgery groups in patients with no previous ALND and negative nodes at time of IBTR (*N* = 72). *LRRFS* locoregional recurrence-free survival, *DMFS* distant metastasis-free survival, *OS* overall survival

As shown in Supplemental Table 2, in the whole cohort, with a median follow-up of 17.5 (IQR range 8.0–38.3) months from IBTR resection, three (1.9%) patients were diagnosed with isolated LRR as the first event in the breast/thoracic wall or supraclavicular node. Notably, eight (5.2%) patients were reported to have LRR with synchronous distant metastasis. Of these, two patients had ipsilateral internal mammary node metastasis with concurrent extensive metastases in the contralateral axilla, five had ipsilateral chest wall recurrence with synchronous distant recurrence, and one had ipsilateral chest wall recurrence with metachronous bone metastasis. Within the 11 patients (7.1%) with a second-LRR, no ipsilateral axillary recurrences were detected. The median time interval from surgery for IBTR to detection of second-LRR for these 11 patients who developed second-LRR was 1.6 years (range: 0.08–4.1). Additionally, twenty (13.0%) distant metastases were noted as the first event, with a median metastasis-free interval of 13 (IQR range 8.0–30.3) months from IBTR removal. The Kaplan–Meier curves of LRRFS, DMFS, and OS based on axillary staging status for IBTR showed no significant difference in survival outcome between the axilla surgery and no axilla surgery groups (Fig. 4A–C). No significant difference was observed in the 2-year LRRFS (93.5% vs 91.0%, *p* = 0.5), DMFS (78.3% vs 79.2%, *p* = 0.76), or OS (93.7% vs 94.6%, *p* = 0.65) rates between the axilla surgery and no axilla surgery groups (Table 3).

**Table 3:** Cox regression analysis of LRRFS, DMFS, and OS after IBTR

Variables	Total patients with IBTR (*N* = 154)			Patients without prior ALND (*N* = 72)		
	No. of events	2-year survival probability (95% CI)	*p* value by log-rank test	No. of events	2-year survival probability (95% CI)	*p* value by log-rank test
LRRFS			0.5			0.77
Axilla surgery	2	93.5 (84.8–100.0)		2	91.6 (80.5–100.0)	
No axilla surgery	9	91.0 (84.1–98.4)		1	91.7 (77.3–100.0)	
DMFS			0.76			0.83
Axilla surgery	9	78.3 (65.4–93.9)		7	80.3 (66.6–96.9)	
No axilla surgery	19	79.2 (69.8–90.0)		4	83.6(67.1–100.0)	
OS			0.65			0.78
Axilla surgery	3	93.7 (85.1–100.0)		2	97.5 (92.8–100.0)	
No axilla surgery	7	94.6 (88.9–100.0)		1	95.7 (87.3–100.0)	

*LRRFS* locoregional recurrence-free survival, *DMFS* distant metastasis-free survival, *OS* overall survival, *IBTR* ipsilateral breast tumor recurrence, *ALND* axillary lymph node dissection, *CI* confidence interval

Additional analysis was performed to evaluate the regional control effect stratified by axillary staging status of primary breast cancer. In patients without previous ALND, with a median follow-up of 21.5 (IQR range 8.5–30) months from IBTR removal, eight (11.1%) patients were observed to have distant relapses, while three patients had ipsilateral chest wall recurrence with synchronous bone or pleural metastases (4.2%), resulting in an overall LRR rate of 4.2% with a 2-year LRRFS of 91.7% (95% CI: 78.9–100.0). Of note, among these 8 patients with re-recurrence, all but 4 underwent completion ALND at time of IBTR. The trend of survival data was consistent with that of the whole cohort in the LRRFS, DMFS, and OS rates (Fig. 4D–F; Table 3) regardless of axillary staging status for IBTR (*p* = 0.77, 0.83 and 0.78, respectively). See Supplemental Table 3 for detailed information regarding the location of recurrences after IBTR and treatment following IBTR. Interestingly, in 38 (52.8%) patients not undergoing completion ALND at time of IBTR, irradiation was offered in 10 (26.3%) patients with no axilla target volume, of which three distant metastases and no regional events were reported during follow-up.

## Discussion

To the best of our knowledge, this is the first study to explore the locoregional control of sparing SAS in IBTR patients. According to our results, the 2-year risk of developing regional recurrence without SAS in patients with IBTR is less than 10%, with no case being located in the ipsilateral axilla, especially in those not undergoing primary ALND. The low relapse rate provides further safe evidence that sparing surgical axillary evaluation could be adopted in the clinical situation of IBTR.

Time intervals between the treatment of primary breast cancer and IBTR provide prognostic information and greatly matter for survival [15, 16]. Later recurrences have consistently been related to more favorable outcomes than earlier recurrences [17]. The recurrence-free interval of this study is 2.6 years, which is significantly shorter than that of the CALOR trial (≥ 5 years) [18]. This might partially be explained by the higher-risk baseline levels of demographics in our cohort, such as younger median age at onset of IBTR (46 years vs 56 years) and higher percentage of chemotherapy for primary tumors (73.4% vs 62.3%).

In the recent past, the performance of ALND in cases of IBTR was considered the standard of care in ipsilateral axillary staging management [19]. Our data demonstrated that the yield of axilla surgery for IBTR was limited, with subclinical nodal involvement detected in 6 of 53 patients (11.3%) receiving ALND or SLNB as part of their axillary staging procedure. In other words, approximately, 87% of patients had pathologically cancer-free nodal status after surgical evaluation, which was comparable to the negative predictive value of preoperative axillary assessment by physical examination and ultrasound in patients treated with SLNB [20]. These results reinforce the available evidence that only a limited portion of the involved lymph nodes has the potential to evolve into clinically detectable axillary disease. Therefore, screening potential patients to avoid SAS for IBTR is an essential focus of current research.

In the early breast cancer scenario, several ongoing prospective randomized trials, such as SOUND, POSNOC, and BOOG 2013-08, are currently comparing SLNB with observation in cN0 patients treated with BCS [20–22]. The results are awaited, but it is clear that the questions addressed in these trials are similar to the questions raised in IBTR. Our study indicated that patients with no previous ALND, longer recurrence interval, discordant molecular subtype, and different recurrence location were more likely to receive axillary evaluation, either ALND or repeat SLNB. However, when refined by prior nodal staging status, no significant differences were observed in the recurrence interval and molecular subtype concordance between the axilla surgery and no axilla surgery groups in patients with no previous ALND. Hence, it is essential to determine a uniform implementation strategy to minimize variations in patterns of care in nodal evaluation on the premise of equivalent regional control.

Few studies have addressed the concerns of refractory locoregional control after the curative treatment of an IBTR. At a median follow-up of 25 months, the second-LRR rate in our study was 7.1% (11/154) with a median time interval of 1.6 years from IBTR resection to re-recurrence diagnosis, which is consistent with that in the CALOR trial (9.0%, 8/89)[23]. Interestingly, a high risk of developing distant diseases rather than axillary recurrence was seen in patients with re-recurrence, wherein the majority of them had ALND either for their primary tumor or for IBTR. The role of ALND in this relatively high-risk group has not been shown to improve survival; therefore, emphasis should be placed on local control of recurrence (not diagnostic procedures, e.g., ALND in a cN0 patient) and therapeutic strategies to decrease the risk of distant recurrence.

Currently, the trends in axillary surgery as a staging tool are decreasing due to customized systemic therapies and the introduction of molecular signatures in clinical decision-making. The results of our study are consistent with the downward trend of ALND in cN0 recurrent patients, as most of the included patients underwent no SAS at time of IBTR surgery (65.6%). Additionally, we found that the extent of axillary involvement did not affect systemic or radiation recommendations. Of interest, in patients not undergoing completion ALND for IBTR and selected for irradiation, the axilla field was not prescribed in clinical target volume, with no regional events reported thereafter. Moreover, excessive axilla surgery could predispose patients to arm lymphedema development, which increases the risk of infections (cellulitis) and fears of cancer recurrence [24]. With the improvement of non-invasive screening methods, such as dedicated axillary lymph node PET (Lymph-PET) [25], it is possible to justify less extensive axillary surgery in node-negative IBTR patients.

Our study has some inherent limitations. First, the retrospective nature of this single-institution-based study carries the risk of bias. Although some prognosis-related factors were comparatively balanced between the two groups at baseline, there might nevertheless be some factors weighing the survival outcome that we neglected. Second, the total number of IBTR patients was comparatively limited. However, in the CALOR trial, along with the NRG Oncology/RTOG 1014 trial, the enrolled numbers of patients with IBTR were only 89 and 65, respectively [4, 18]. Both sample sizes allowed for continued analyses. Finally, the second recurrence events after curative treatment of IBTR were also small because of inadequate follow-up duration. The results of the study need to be reinforced by a long-term follow-up period and more cumulative events.

In conclusion, for node-negative IBTR patients, we observed selection bias on the basis of prior ALND, shorter recurrence interval, concordant molecular subtype, and same quadrant location favoring no surgical nodal staging but comparable LRRFS, DMFS, and OS. These results support a wider consideration of sparing SAS in the management of IBTR, especially in the setting of patients without previous ALND.

## Supplementary Information

Below is the link to the electronic supplementary material.

Supplementary file1 (TIF 8989 KB) Figure S1. Transitions between clinicopathologic features from primary tumor to IBTR. *IBTR* ipsilateral breast tumor recurrence, *IHC* immunohistochemistry, *HR* hormone receptor

Supplementary file2 (DOCX 23 KB)

## Data Availability

The datasets generated during and/or analyzed during the current study are available from the corresponding author on reasonable request.

## Abbreviations

ALND: Axillary lymph node dissection
BCS: Breast-conserving surgery
cN +: Clinically positive axillary lymph node
cN0: Clinically negative axillary lymph node
DMFS: Distant metastasis-free survival
EBC: Early-stage breast cancer
IBTR: Ipsilateral breast tumor recurrences
LRR: Loco-regional recurrence
LRRFS: Loco-regional recurrence-free survival
OS: Overall survival
SAS: Surgical axillary staging
SLNB: Sentinel lymph node biopsy
