# Supplementary figures and images for: Omission of axillary surgery for ipsilateral breast tumor recurrence with negative nodes after previous breast-conserving surgery: is it oncologically safe?
Source: Breast Cancer Res Treat. 2022 Aug 30;196(1):97–109. doi: 10.1007/s10549-022-06708-y (PMC9550716; doi:10.1007/s10549-022-06708-y)

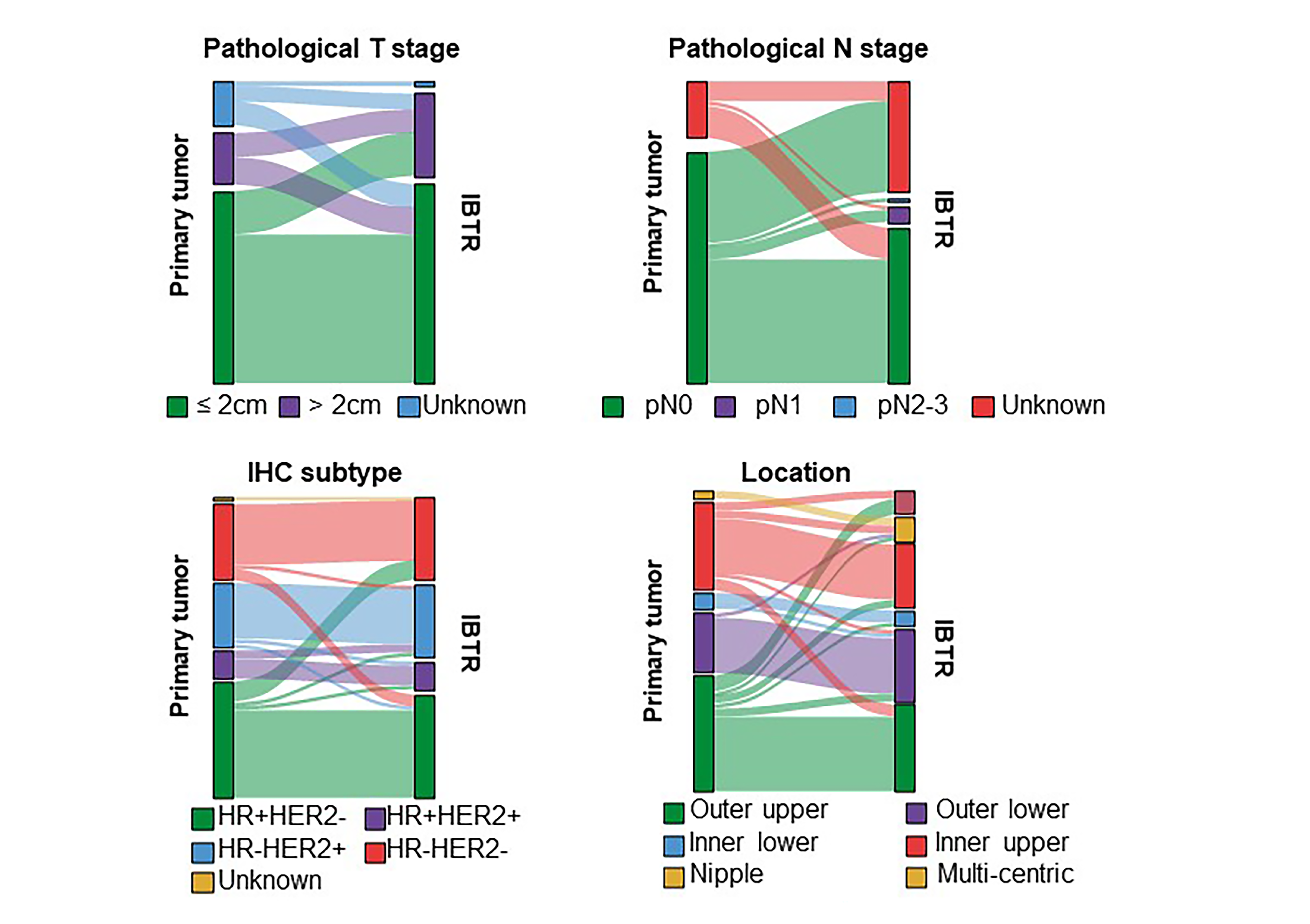

Supplement: Supplementary file 1 — Supplementary file1 (TIF 8989 KB) Figure S1. Transitions between clinicopathologic features from primary tumor to IBTR. IBTR ipsilateral breast tumor recurrence, IHC immunohistochemistry, HR hormone receptor [file 10549_2022_6708_MOESM1_ESM.tif]
