# Supplementary material for: Omission of axillary surgery for ipsilateral breast tumor recurrence with negative nodes after previous breast-conserving surgery: is it oncologically safe?
Source: Breast Cancer Res Treat. 2022 Aug 30;196(1):97–109. doi: 10.1007/s10549-022-06708-y (PMC9550716; doi:10.1007/s10549-022-06708-y)
Supplement: Supplementary file 2 — Supplementary file2 (DOCX 23 KB) [file 10549_2022_6708_MOESM2_ESM.docx]

**Supplementary Table 1. Identification of repeat sentinel node biopsy**

| **No.** | **Primary axillary staging** | **Primary SNB/AND number** | **rSNB location surgical harvested** | **rSNB number** |
| --- | --- | --- | --- | --- |
| **P1** | SNB negative | 3 | Ipsilateral axilla | 4 |
| **P2** | SNB negative | 4 | Ipsilateral axilla | 5 |
| **P3** | SNB negative | 4 | No SN | N/A |
| **P4** | SNB negative | 3 | Ipsilateral axilla | 4 |
| **P5** | SNB negative | 2 | Ipsilateral axilla | 5 |
| **P6** | AND pN0 | 16 | No SN | N/A |
| **P7** | AND pN1 | 14 | Ipsilateral axilla | 5 |

Abbreviations: *SNB*, sentinel node biopsy; *AND*, axillary node dissection; *rSNB*, repeat sentinel node biopsy; *SN*, sentinel node.

**Supplementary Table 2. Regional and distant recurrences as first event after curative treatment of IBTR (N=154)**

| **Rerecurrence events (N=31)** | **Number** | **Incidence (%)** |
| --- | --- | --- |
| **Locoregional** | 3 (9.7%) | 1.9 |
| In-breast | 1 |  |
| Chest wall and skin | 1 |  |
| SCLN | 1 |  |
| **Distant metastasis** | 20 (64.5%) | 13.0 |
| Bones | 7 |  |
| Lung | 4 |  |
| Liver | 1 |  |
| Brain | 3 |  |
| Others | 5 |  |
| **Locoregional+ distant metastasis** | 8 (25.8%) | 5.2 |
| IMN + contralateral ALN | 2 |  |
| Chest wall + lung/liver/brain metastasis | 5 |  |
| Chest wall + bone metastasis* | 1 |  |

**Abbreviations:** *IBTR*, ipsilateral breast tumor recurrence; *SCLN*, supraclavicular lymph node; *ALN*, axillary lymph node.

*Metachronous.

**Supplementary Table 3. Regional and distant recurrences as first event after curative treatment of IBTR in patients with no prior ALND**

**(N=72)**

| **No** | **Location of Recurrence** | **Follow-up IBTR (months)** | **DFI**  **(months)** | **Primary axillary staging** | **IBTR**  **axillary staging** | **Primary adjuvant (CT, RT, EnT)** | **Secondary adjuvant**  **(CT, RT, EnT)** |
| --- | --- | --- | --- | --- | --- | --- | --- |
| P1 | Ipsilateral chest wall; multiple bone metastasis | 17 | 37 | SLN- | None | CT (ddEC*4), RT | CT (Xol) |
| P2 | Ipsilateral chest wall;  bilateral 3^rd^ anterior ribs | 6 | 40 | SLN- | ALND- | CT (CEF-T+H), RT | CT (EC-T+H) |
| P3 | 5^th^ lumbar vertebral | 4 | 63 | SLN- | ALND- | CT (T-CEF) | CT (ddPtx*6), RT |
| P4 | Regional-distant LN | 10 | 7 | SLN- | ALND- | CT (EC-Ptx) | CT (GPtx*4) |
| P5 | Frontal lobe | 28 | 12 | SLN- | ALND- | CT (CEF), RT | CT (TC*4), EnT (Ana) |
| P6 | Skull | 4 | 13 | SLN- | ALND- | CT (ddEC-Ptx), RT | CT (Xol), BiPH |
| P7 | Liver | 13 | 26 | SLN- | ALND- | CT (ET), RT | CT (NVB+H) |
| P8 | 12^th^ thoracic vertebral | 35 | 50 | SLN- | None | None | RT, EnT (Exe) |
| P9 | Contralateral axilla | 7 | 62 | SLN- | ALND- | CT (CEF-T), RT | CT (Xol+H+P), EnT (AI) |
| P10 | Multiple bone metastasis; distant LN | 14 | 13 | None | None | CT (EC-wP) | CT (NVB+Xol), RT |
| P11 | Ipsilateral chest wall; pleural effusion | 34 | 42 | None | None | CT (CEF-T), RT, EnT (TAM) | CT (NVB+Xol+H),  EnT (TOR) |

**Abbreviations:** *IBTR*, ipsilateral breast tumor recurrence; *DFI*, disease-free interval; *CT*, chemotherapy; *RT*, radiotherapy; *EnT*, endocrine therapy; *LN*, lymph node; *SLN-*, sentinel lymph node negativity; *ALND-*, axillary lymph node dissection negativity; *dd*, dose-dense; *E*, epirubicin; *C*, cyclophosphamide; *F*, 5-fluorouracil; *T*, docetaxel; *H*, trastuzumab; *Ptx*, paclitaxel; *Xol*, capecitabine; *G*, gemcitabine; *Ana*, anastrozole; *BiPH*, bisphosphonates; *NVB*, vinorelbine; *Exe*, exemestane; *P*, pertuzumab; *TAM*, tamoxifene; *TOR*, toremifene.
